# Identification of far-red light acclimation in an endolithic *Chroococcidiopsis* strain and associated genomic features: Implications for oxygenic photosynthesis on exoplanets

**DOI:** 10.3389/fmicb.2022.933404

**Published:** 2022-08-04

**Authors:** Daniela Billi, Alessandro Napoli, Claudia Mosca, Claudia Fagliarone, Roberto de Carolis, Amedeo Balbi, Matteo Scanu, Vera M. Selinger, Laura A. Antonaru, Dennis J. Nürnberg

**Affiliations:** ^1^Department of Biology, University of Rome Tor Vergata, Rome, Italy; ^2^Ph.D. Program in Cellular and Molecular Biology, Department of Biology, University of Rome Tor Vergata, Rome, Italy; ^3^Department of Physics, University of Rome Tor Vergata, Rome, Italy; ^4^Department of Physics, Biochemistry and Biophysics of Photosynthetic Organisms, Freie Universität Berlin, Berlin, Germany

**Keywords:** FaRLiP, desert cyanobacteria, *Chroococcidiopsis*, astrobiology, biosignature

## Abstract

Deserts represent extreme habitats where photosynthetic life is restricted to the lithic niche. The ability of rock-inhabiting cyanobacteria to modify their photosynthetic apparatus and harvest far-red light (near-infrared) was investigated in 10 strains of the genus *Chroococcidiopsis*, previously isolated from diverse endolithic and hypolithic desert communities. The analysis of their growth capacity, photosynthetic pigments, and *apcE2*-gene presence revealed that only *Chroococcidiopsis* sp. CCMEE 010 was capable of far-red light photoacclimation (FaRLiP). A total of 15 FaRLiP genes were identified, encoding paralogous subunits of photosystem I, photosystem II, and the phycobilisome, along with three regulatory elements. CCMEE 010 is unique among known FaRLiP strains by undergoing this acclimation process with a significantly reduced cluster, which lacks major photosystem I paralogs *psaA* and *psaB*. The identification of an endolithic, extremotolerant cyanobacterium capable of FaRLiP not only contributes to our appreciation of this phenotype’s distribution in nature but also has implications for the possibility of oxygenic photosynthesis on exoplanets.

## Introduction

The discovery of cyanobacteria capable of harvesting far-red light (near-infrared, wavelengths > 700 nm) has changed the paradigm that oxygenic photosynthesis is only driven by visible light and exclusively by chlorophyll *a* (Chl *a*) (Chen et al., 2012; Loughlin et al., 2013; Gan et al., 2014). The red limit of photosynthesis is of particular interest to astrobiology. M-stars, the most common type of star in the galaxy, have a light spectrum that peaks in the far-red and infrared. Some may be orbited by Earth-like planets, and any photosynthetic life potentially inhabiting them would likely use these wavelengths (Cockell et al., 2009). Furthermore, it is well known that pigments involved in oxygenic photosynthesis exhibit a sharp increase in reflectance at near-infrared wavelengths, a feature usually termed “red edge,” that can be used as a remote indication of life on rocky planets occurring in the so-called habitable zone (see, e.g., Hegde and Kaltenegger, 2013).

On Earth, far-red light photosynthesis occurs in cyanobacteria living in environments where visible light is strongly attenuated, for instance, by competing for photosynthetic organisms or by physical conditions (Larkum and Kühl, 2005; Gan and Bryant, 2015). This creates selective pressure for using photons that are normally too low in energy to drive water splitting. There are two known types of far-red oxygenic photosynthesis: A constitutive adaptation that uses a majority of chlorophyll *d* and is restricted to a single genus (*Acaryochloris*), and an acclimation response (far-red light photoacclimation, or FaRLiP), which is present in phylogenetically diverse cyanobacteria (Gan and Bryant, 2015; Averina et al., 2018; Antonaru et al., 2020). The latter uses ∼10% chlorophyll *f*, alongside a majority of chlorophyll *a* and traces of chlorophyll *d* (Gan et al., 2014; Nürnberg et al., 2018). Both chlorophylls *f* and *d* are substituted, spectrally red-shifted variants of chlorophyll *a.*

Chlorophyll *d* was first discovered > 40 years ago (Manning and Strain, 1943), and recent 16S rRNA and pigment analysis data suggest a potential global distribution (see, e.g., Kashiyama et al., 2008; Behrendt et al., 2014; Zhang et al., 2019). In contrast, the first Chl *f*-containing species, *Halomicronema hongdechloris*, has been isolated only within the last decade from stromatolites in Shark Bay, Western Australia (Chen et al., 2012). Since then, more Chl *f*-producing cyanobacteria have been found growing under selective far-red light samples collected from caves, lakes, beach rocks, hot spring mats, and subtropical forests (Akutsu et al., 2011; Behrendt et al., 2015; Trampe and Kühl, 2016; Ohkubo and Miyashita, 2017; Goìmez-Lojero et al., 2018; Zhang et al., 2019; Kühl et al., 2020). In addition, a wealth of data has come from sequence-based searches. Many of the known Chl *f*-producing cyanobacteria were identified by searching publicly available genomes for a gene cluster associated with far-red light photoacclimation (FaRLiP) (Gan et al., 2014, 2015). In addition, insights into the distribution of Chl *f*-photosynthesis at a global scale were later provided by screening environmental metagenomic data with a single far-red gene as a marker (Antonaru et al., 2020). This sequence (*apcE2*) encodes the core-membrane linker of the far-red phycobilisome. Overall, the FaRLiP cluster consists of ∼19 genes encoding paralogous subunits of the photosystem I (PSI), photosystem II (PSII), and the phycobilisome (PBS), along with three *rfp* genes encoding master control elements (Gan et al., 2014, 2015; Zhao et al., 2015; Wiltbank and Kehoe, 2019). A highly divergent PSII paralog, *chlF*, also known as *psbA4*, is involved in chlorophyll *f* synthesis (Ho et al., 2016; Trinugroho et al., 2020). These genes are most commonly found together but may occasionally be split into two subclusters (Gan et al., 2015; Sheridan et al., 2020).

The FaRLiP capacity of rock-inhabitant cyanobacteria in extreme environments has been poorly investigated. There has been one report focusing on the subject (Murray et al., 2022), together with one report on the far-red *apcE2* gene being present in the metagenome of endolithic communities from the Atacama Desert (Antonaru et al., 2020). In fact, in hot and cold deserts, cyanobacteria take refuge in or under rocks, where the transmitted light is still sufficient for photosynthesis (Cockell et al., 2009). Different rock substrates have different light transmissions, for instance, calcite is more translucent than granite, gypsum, and ignimbrite, although they all have a higher transmission of red wavelengths compared to blue (Smith et al., 2014; Meslier et al., 2018). Depending on the mineralogical composition, cyanobacterial colonization can be endolithic, including beneath the rock surface within pores (cryptoendoliths) or in cracks or fissures (chasmoendoliths), but also hypolithic, at the rock–soil interface (Wierzchos et al., 2011). Recently, chlorophylls *d* and *f* were reported for endolithic communities from gypsum, calcite, and sandstone from the Atacama and Namib deserts (Murray et al., 2022), but these pigments were not detected in hypolithic communities from quartz pebbles in the latter desert (Gwizdala et al., 2021). Red-shift carotenoids were also reported for endolithic communities (primarily composed of *Chroococcidiopsis* cyanobacteria) in ignimbrite rocks from the Atacama Desert (Vítek et al., 2017). Moreover, a red-shifted emission spectrum of the photosynthetic pigments was reported for *Chroococcidiopsis* cells occurring in hypolithic communities in quartz, carbonate, and talc from the Mojave Desert (Smith et al., 2014). This raises the possibility that FaRLiP may be significantly present.

In this study, the FaRLiP capacity of rock-inhabiting cyanobacteria was investigated in 10 strains of *Chroococcidiopsis* isolated from endolithic and hypolithic communities collected from five deserts worldwide. The selected strains were grown under far-red light (FRL) and white light (WL) and tested for growth, photosynthetic pigment features (as revealed by confocal laser scanning microscopy), and the presence of the far-red *apcE2* gene (by polymerase chain reaction). Among the 10 investigated strains, only *Chroococcidiopsis* sp. CCMEE 010 showed a FaRLiP response. Therefore, the Oxford Nanopore and Illumina platforms were used to obtain the whole-genome sequence of this strain, and a bioinformatics search was performed to identify FaRLiP-relevant genes. Finally, to evaluate the Chl *f* detectability as a far-red shifted signature of life on exoplanets, the reflectance spectrum of dried cells of this far-red positive *Chroococcidiopsis* strain was measured and compared to that of a negative far-red *Chroococcidiopsis* strain.

## Materials and methods

### Strains and growth conditions

A total of 10 *Chroococcidiopsis* strains were obtained from the Culture Collection of Microorganisms from Extreme Environments (CCMEE) established by E. Imre and Roseli Ocampo-Friedmann and currently maintained at the Department of Biology, University of Tor Vergata (Table 1). White light (WL) was provided by cool white fluorescent bulbs and FRL by 750 nm LEDs (Epitex; L750-01AU). Triplicates were grown for 14 days in liquid BG-11 medium at 25°C under permanent WL and FRL illumination with a photon flux density of approximately 40 μmol m^–2^ s^–1^ for both light sources. After a 14-day incubation, the growth was evaluated by measuring the optical density (OD) at 730 nm using a spectrophotometer.

**TABLE 1:** List of *Chroococcidiopsis* strains used in this study.

CCMEE strain	Sampling site	Rock substrate/ colonization type
06	Negev Desert, Israel	Sandstone/cryptoendolithic
07	Negev Desert, Israel	Granite/cryptoendolithic
010	Negev Desert, Israel	Granite/chasmoendolithic
019	Negev Desert, Israel	Calcite/chasmoendolitic
046	Negev Desert, Israel	Limestone/hypolithic
053	Sinai Desert, Egypt	Granite/chasmoendolitic
064	Sinai Desert, Egypt	Stone pavement/hypolithic
078	Mojave Desert, California	Granite/chasmoendolitic
088	Sonora Desert, Mexico	Granite/chasmoendolithic
313	Broken Hill Desert, Australia	Granite/hypolithic

### Confocal laser scanning microscopy

Culture aliquots of the selected 10 *Chroococcidiopsis* strains grown for 14 days under WL and FRL were immobilized onto a BG-11 medium containing 1.5% agarose and examined with a Confocal Laser Scanning Microscopy System (CLSM; Olympus Fluoview 1000). CLSM lambda scans were obtained by using a 488 nm excitation laser (and collecting the emission from 550 to 800 nm). Curve plotting was performed using the GraphPad Prism program (GraphPad Software, San Diego, CA). Fluorescence images of *Chroococcidiopsis* sp. CCMEE 010 cells were acquired with a Leica SP8 inverted CLSM by exciting the immobilized cells with a 488 nm laser and acquiring the fluorescence emission from 650 to 680 nm for phycobilisomes and Chl *a*, and from 720 to 750 nm for Chl *f*.

### Reflectance measurement

Liquid-culture aliquots of *Chroococcidiopsis* were immobilized on 0.2-μm polycarbonate filters (Millipore, Burlington, MA, United States), dried under a laminar flow hood for 24 h, and then stored at RT in the dark. Reflectance spectra were acquired with an Agilent Cary 60 Remote Diffuse Reflectance Accessory connected to an Agilent Cary 60 UV/Vis spectrometer.

### PCR amplification of far-red specific genes

The genomic DNA of the ten *Chroococcidiopsis* sp. strains was extracted using a PowerWater DNA Isolation Kit (MO BIO Laboratories, Carlsbad, CA, United States) and quantified using the NanoDrop Lite Spectrophotometer (Thermo Fisher Scientific, Waltham, MA, United States). Amplification of *apcE2* was performed by using 12 ng of genomic DNA in 12 μl PCR reaction mixtures containing 6 μl of Phusion High-Fidelity PCR Master Mix with HF Buffer (New England Biolabs, Ipswich, MA, United States) and F-apcE2M and R-apcE2M primers as described (Antonaru et al., 2020). The resulting amplicons were loaded onto a 1.5% agarose gel containing 0.5 mg ml^–1^ ethidium bromide and, after electrophoresis, visualized with a transilluminator.

For *psaA2* and *psaB2*, PCR involved 50 ng of gDNA in 25 μl reactions, using a Q5 High-Fidelity 2X Master Mix (New England Biolabs, Ipswich, MA, United States). Primers are listed in Supplementary Table 1. Thermocycling conditions were as reported above, except for a 1 min extension time, and a gradient annealing temperature as described below. The results were visualized on a 1.5% agarose gel stained with ROTI GelStain Red (Carl Roth GmbH, Karlsruhe, Germany).

### Identification of far-red genes in CCMEE 010’s genome

Genomic DNA of *Chroococcidiopsis* sp. CCMEE 010 was sequenced using Illumina MiSeq and Oxford Nanopore MinION. Illumina libraries were prepared using the Kapa Hyperplus library kit (Roche Molecular System Inc.) following the manufacturer’s instructions. The final pooled library was quantified by qPCR and sequenced using the MiSeq Reagent Kit V3. The Oxford Nanopore libraries were prepared following the manufacturer’s instructions. Samples were first labeled using a rapid barcoding kit (SQK-RBK004) and then sequenced through the ligation kit LSK-SQK109. Base calls were performed using the guppy software 4.4.1.^1^ Illumina and Nanopore reads were assembled together using Unicycler version 0.4.8 (Wick et al., 2017), a hybrid assembly pipeline for bacterial genomes. The assembly yielded a circular genome (length 5,449,630 bp, mean coverage ∼113x, with a minimum of 41x) that was annotated with PROKKA version 1.14.5 (Seemann, 2014) through the interface provided by Galaxy (Afgan et al., 2018). Putative FaRLiP genes were recovered and confirmed by using BLAST, e.g., against the NCBI nr database (Johnson et al., 2008; Sayers et al., 2021).

### Multiple sequence alignment

Cyanobacterial far-red genes were downloaded from GenBank (National Centre for Biotechnology Information, NCBI) and Uniprot databases (Uniprot, 2021), and multiple alignments were obtained by using Clustal Omega (Sievers and Higgins, 2014). Sequence alignments were displayed graphically using JalView 2.10.5 (Waterhouse et al., 2009).

### Confirmation of gene absence by PCR

Initial searches revealed the absence of far-red *psaA* and *psaB* paralogs in both assembled and unassembled data. Due to the fragmented nature of the latter, short (19–22 aa), FaRLiP-specific motifs were used as queries (*psaA*: AQPLGDVFGGVRGIELSGLGTT; *psaB*: LVWAHEKTPLSFGYWRDKP). For the validation of the gene loss, PCR primers were developed that could preferentially amplify these genes from genomic material. Both the conserved motifs and the primers targeting them were built by comparing far-red *psaA* and *psaB* sequences using the species dataset from Antonaru et al. (2020) with standard, white-light paralogs (recovered by BLAST from NCBI). To build the motifs, 60–70 sequences were used per paralog per gene. Primers were built using the Multiple Primer Analyzer web tool (Thermo Fisher Scientific, Waltham, MA, United States). The variation within FaRLiP sequences, combined with the high sequence similarity to standard paralogs, limits the usefulness of these primers to closely related clades (Chroococcidiopsidales and some heterocyst-forming cyanobacteria). For *psaA*, primers could not accurately distinguish between FaRLiP and WL paralogs. Nevertheless, the amplicons could be distinguished by size. A 70–80 bp insertion is specific to FaRLiP *psaA* in all sequences studied apart from the distantly related *Leptolyngbya* sp. JSC-12. Amplicons were checked by sequencing (Microsynth, Balgach, Switzerland).

## Results

### *Chroococcidiopsis* CCMEE 010 has the capacity for far-red light photoacclimation

**FIGURE 1:**
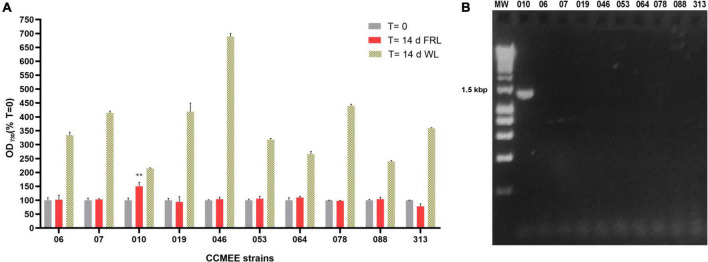
Capacity for photosynthesis under far-red light in 10 desert *Chroococcidiopsis* strains. **(A)** Cell densities after 14-day incubation under WL or FRL; values at time zero are set as 100. All WL cultures showed a significant increase in OD_730_ (*p*-value < 0.001, not shown) after 14 days under FRL a significant increase in OD_730_ occurred only in CCMEE 010 (^**^p-value < 0.01). **(B)** PCR amplification of the far-red *apcE2* gene. MW, molecular weight (HyperLadder 1 kb, Bioline Inc., MA, United States).

A total of 10 *Chroococcidiopsis* strains isolated from endolithic and hypolithic communities from five different deserts (Table 1) were grown under WL and FRL (Figure 1A). After 14 days of FRL incubation, only strain CCMEE 010 showed an increase in cell density compared to time zero. No increase in cell density was observed in the other nine strains, namely, CCMEE 06, 07, 019, 046, 053, 064, 078, 088, and 313 (Figure 1A). All the ten strains showed increased cell densities when grown under white light (Figure 1B).

When the 10 *Chroococcidiopsis* strains were screened for the presence of the far-red marker gene *apcE2*, a PCR amplicon of the expected size of ∼1.2 kbp was obtained only for CCMEE 010 (Figure 1B).

### *Chroococcidiopsis* CCMEE 010 shows spectral features associated with Chl *f* formation

**FIGURE 2:**
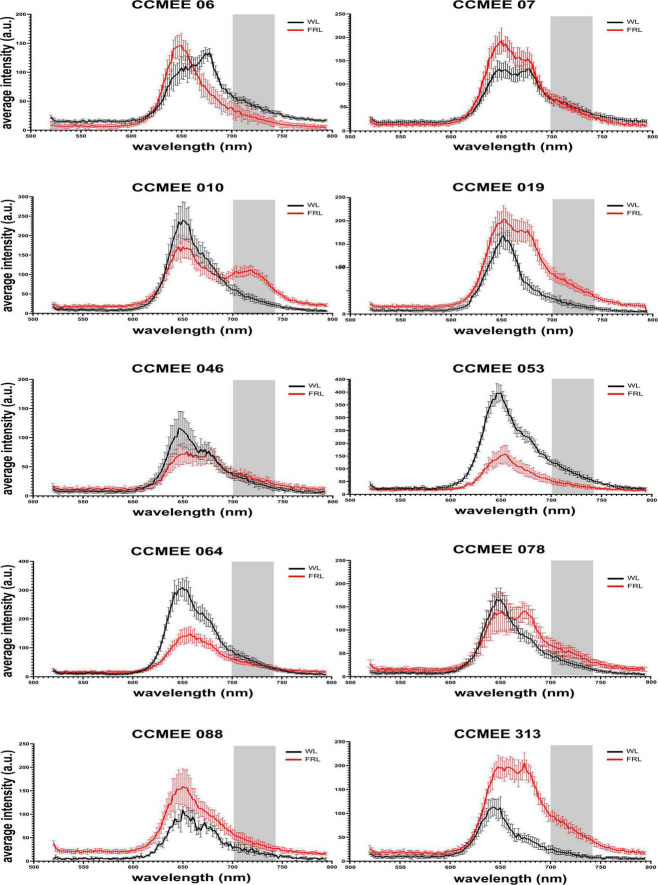
CSLM spectral scans of individual cells of FaRLiP negative *Chroococcidiopsis* spp. CCMEE strains grown for 14 days under white or far-red illumination.

**FIGURE 3:**
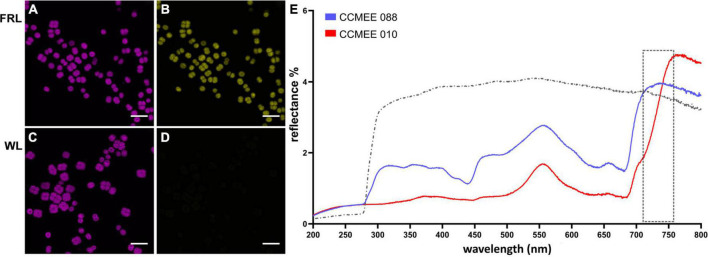
Spectral characteristics of far-red light photoacclimation. CSLM images of *Chroococcidiopsis* sp. CCMEE 010 grown for 14 days under FRL **(A)** or WL **(C)** in the 650–680 nm emission range. Red-shifted Chl *f* presence in FRL-acclimated cells in the 720–750 nm emission range **(B)** and absence in WL cells **(D)**. Scale bars 20 μm. Reflectance spectra **(E)** of the FaRLiP positive *Chroococcidiopsis* sp. CCMEE 010 (red line) and FaRLiP negative *Chroococcidiopsis* sp. CCMEE 088 (blue line) dried on polycarbonate filters (gray line).

All *Chroococcidiopsis* strains under study showed fluorescence emission peaks in the range of 645–680 nm under white light. This can be attributed to phycobiliproteins and Chl *a*-containing photosystems. Nine of these strains (namely, CCMEE 06, 07, 019, 046, 053, 064, 078, 088, and 313) also maintained this emission range under far-red light but showed some variation in the intensity associated with a change in phycobilisome and photosystem content (Figure 2). A single strain, *Chroococcidiopsis* sp. CCMEE 010 (Figure 2), showed an additional peak around 715–725 nm under FRL. This is a characteristic of the red-shifted Chl *f*, and it has been previously used to identify cyanobacteria capable of FaRLiP (Chen et al., 2012). The slightly shifted fluorescence emission of Chl *f* to shorter wavelengths and the reduction in the overall fluorescence intensity in comparison to previous studies might be caused by the decreased sensitivity of the detector above 700 nm, in fact, the fluorescence in the region from 720 to 750 nm is shown in Figure 3B.

The difference in fluorescence emission spectra was also used for CLSM imaging. *Chroococcidiopsis* sp. CCMEE 010 cells grown under FRL showed the emission of phycobilisomes and Chl *a* (Figure 3A, 650–680 nm) as well as of Chl *f* (Figure 3B, 720–750 nm). In contrast, WL-grown cells showed Chl *a* emission (Figure 3C) but lacked the Chl *f* emission (Figure 3D), similar to what was observed for closely related *Chroococcidiopsis thermalis* PCC 7203 (MacGregor-Chatwin et al., 2022).

Spectral differences are also evident in reflectance measurements. Dried samples of FRL-grown cells of *Chroococcidiopsis* sp. CCMEE 010 had a spectrum extended by 50 nm into the far red and a reduced signal in the blue range when compared to FaRLiP negative *Chroococcidiopsis* sp. CCMEE 088 (Figure 3E). The shift in the so-called “red edge” indicated a different pigment composition and the presence of far-shifted Chl *f* occurring after 14 days of FRL incubation in CCMEE 010 cells (Figure 3E).

### Unusual genetic basis of far-red light photoacclimation

**FIGURE 4:**
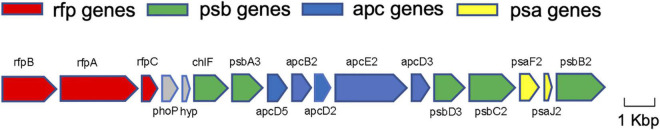
Far-red genes of *Chroococcidiopsis* sp. CCMEE 010. Color-coding for genes: *psa* genes for subunits of PSI (yellow); *psb* genes of PSII (green); *apc* genes of PBS (blue); *rfp* genes for the knotless phytochrome (RfpA) and response regulators RfpB and RfpC (red); genes presumed unrelated to photosynthesis are shown in gray. The figure was built using Gene Graphics (Harrison et al., 2018).

Out of nineteen FaRLiP genes expected, fifteen were identified in the genome assembly of CCMEE 010 by using homology and synteny information (Table 2). These include two PSI paralogs (*psaF* and *psaJ*), three components of a phytochrome signaling cascade (*rfpA*/*B*/*C*), five genes encoding for phycobilisome components (*apcE2, apcD2*/*3*/*5*), and five PSII paralogs (*psbA3, psbA4, psbB2, psbC2*, and *psbD3*). Of the latter, *psbA4*, also known as *chlF* or super-rogue, is a highly divergent D1 gene, which does not form an oxygen-evolving PSII complex but rather is involved in Chl *f* synthesis (Ho et al., 2016; Trinugroho et al., 2020). All these genes were found in a single cluster (Figure 4).

**TABLE 2:** Far-red cluster genes in *Chroococcidiopsis* sp. CCMEE 010’s genome (in red missing genes).

FaRLiP genes	Gene product according to PROKKA annotation	GenBank accession number
PSI		
*psaF2*	Photosystem I reaction center subunit III	OM156478
*psaJ2*	Photosystem I reaction center subunit IX	OM156479
*psaA2*	Chlorophyll *a* apoprotein A1	
*psaB2*	Chlorophyll *a* apoprotein A2	
*psaI2*	Photosystem I reaction center subunit VIII	
*psaL2*	Photosystem I reaction center subunit IX	
PSII		
*psbA4* (*chlF*)	Chlorophyll *f* synthase	OM156469
*psbA3*	Photosystem II protein D1	OM156470
*psbD3*	Photosystem II protein D2	OM156476
*psbC2*	CP43 reaction center protein	OM156477
*psbB2*	CP47 reaction center protein	OM156480
*psbH2*	Photosystem II peripheral protein H*	
PBS		
*apcD5*	Allophycocyanin subunit alpha-B	OM156471
*apcB2*	Allophycocyanin beta chain	OM156472
*apcD2*	Allophycocyanin subunit alpha-B	OM156473
*apcE2*	Phycobiliprotein core-membrane linker	OM156474
*apcD3*	Allophycocyanin subunit alpha-B	OM156475
Master control		
*rfpA*	Knotless phytochrome with a kinase domain	OM156467
*rfpB*	Transcriptional activator	OM156466
*rfpC*	Response regulatory protein	OM156468

*Not present in all cyanobacteria.

Four photosystem I paralogs, present in all other strains known to be capable of FaRLiP, are absent from the CCMEE 010 cluster. These include major components *psaA* (∼2,350 bp) and *psaB* (∼2,225 bp), as well as minor components *psaL* (∼550 bp) and *psaI* (∼150 bp). They were not found by BLAST searches in the rest of the assembled contigs. As it is not unusual for paralogs to break assemblies, conserved far-red motifs for *psaA* and *psaB* were also used to search unassembled data, but unsuccessfully. Genes *psaI* and *psaL* were considered too small and variable for this task.

The high coverage of the assembly made it unlikely that the genes had been missed in the sequencing. As such, variable primers were built to test for the presence of far-red *psaA* and *psaB* in genomic DNA (Supplementary Figure 1A). Far-red specific amplicons were obtained from the related strain *Chroococcidiopsis thermalis* PCC 7203, as well as the less-closely related heterocyst-forming cyanobacterium *Chlorogloeopsis fritschii* PCC 6912 (Supplementary Figure 1B). However, they were absent from CCMEE 010. No amplicons at all were recovered with *psaB* FaRLiP-specific primers from CCMEE 010 or from the negative control *Acaryochloris marina* MBIC11017 (which lacks FaRLiP). Only a standard WL *psaA* amplicon was recovered from CCMEE 010 using the *psaA* primers.

### Conserved far-red light molecular markers

**FIGURE 5:**
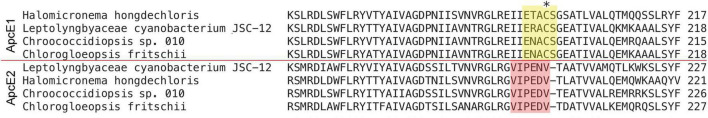
Alignment of ApcEs from *Chroococcidiopsis* sp. CCMEE 010 with homologs from FaRLiP cyanobacteria. Conserved ENACS-motif of ApcE1 is shaded yellow, asterisk (*) shows phytochrome-binding cysteine, VIPEDV-motif of ApcE2 is shaded light red according to Antonaru et al. (2020). UniProtKB: A0A1Z3HIK5_9CYAN, A0A1Z3HPW3_9CYAN; K8GCZ7_9CYAK, K8GJS2_9CYAN; A0A3S0YF90_CHLFR, A0A433NA38_CHLFR.

A total of two *apcE* phycobilisome-membrane linker sequences were recovered from the assembly. To confirm which of them is the FaRLiP version, they were translated and aligned with FR (ApcE2) and standard WL (ApcE1) sequences from *Halomicronema hongdechloris* C2206, *Leptolyngbyaceae* cyanobacterium JSC-12, and *Chlorogloeopsis fritschii* PCC 6912 (Figure 5). Standard paralogs are characterized by an ENACS-like motif that includes phytochrome-binding cysteine, while the FaRLiP proteins bind the phytochrome non-covalently using a VIPEDV-like motif (Gan et al., 2015; Antonaru et al., 2020). The FaRLiP sequence from *Chroococcidiopsis* sp. CCMEE 010 is present in the FaRLiP cluster, with the additional version being characteristic of WL. As previously observed for other strains, the FaRLiP sequence is considerably shorter than the WL one (774 aa as opposed to 1,133 aa), due to the presence of only two (as opposed to four) REP domains (Gan et al., 2015).

**FIGURE 6:**
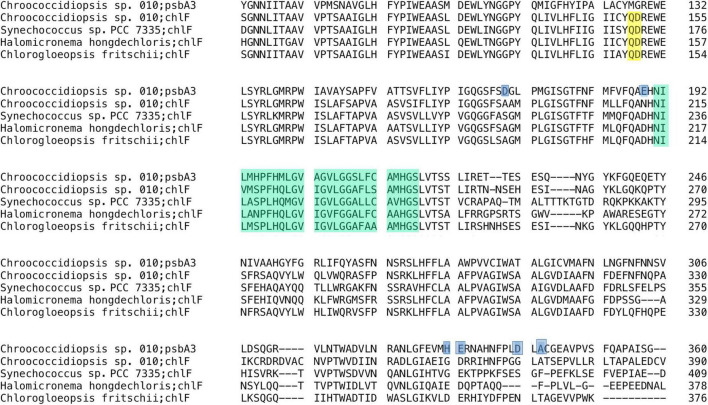
Alignment of ChlF from *Chroococcidiopsis* sp. CCMEE 010 with homologs from FaRLiP cyanobacteria. ChlFs lack ligands to the Mn4CaO5 oxygen-evolving center shaded blue in the PsbA3 sequence of CCMEE 010 and show the conserved QD residues shaded yellow. Photosynthetic reaction center proteins signature (PROSITE entry PS00244) is shaded green. UniProtKB: P0DOC9 (CHLF_CHLFP); B4WP19 (CHLF_SYNS7); A0A1Z3HIN9 (A0A1Z3HIN9_9CYAN).

It could be argued that a more direct marker for FaRLiP is *chlF* (super rogue/*psbA4)*, the synthase for red-shifted chlorophyll *f* (Ho et al., 2016; Trinugroho et al., 2020). In particular, two amino acids (the QD site) were shown to be fully conserved, as well as necessary and sufficient for this synthase function (Trinugroho et al., 2020). The *chlF* sequence from *Chroococcidiopsis* sp. CCMEE 010 showed this site (Figure 6, residues 150–151). In addition, the ChlF of *Chroococcidiopsis* sp. CCMEE 010 lacked key residues required for binding the Mn_4_CaO_5_ cluster for water oxidation as previously reported (Murray, 2012; Ho et al., 2016), which were conversely present in the PsbA3 paralog (Figure 6). This protein is encoded by the gene adjacent to *chlF* in the FaRLiP cluster and contains residues for key cofactor binding for water oxidation and charge separation under FRL conditions (Figure 4).

## Discussion

Out of ten *Chroococcidiopsis* strains, isolated from varied microenvironments in five different deserts, only strain CCMEE 010 was capable of FaRLiP. It showed a far-red shift in the emission spectrum of its photosynthetic pigments under far-red illumination. Furthermore, PCR showed the presence of the far-red *apcE* gene, used as a marker for chlorophyll *f*-producing cyanobacteria (Antonaru et al., 2020).

This prompted a more in-depth analysis by whole-genome sequencing. A total of 15 FaRLiP genes were identified in a cluster. Their sequence similarity with homologous FaRLiP sequences, together with the fact that they include conserved far-red associated motifs, suggests they are functional. However, in terms of gene arrangement, the *Chroococcidiopsis* sp. CCMEE 010 far-red cluster is distinct not only from the cluster in the related *Chroococcidiopsis thermalis* PCC 7203 but also from clusters in more distantly related strains such as the heterocyst-forming cyanobacteria (Supplementary Figure 2). A *Chroococcidiopsis*-associated cluster recovered from an Atacama metagenome also did not show these changes (Murray et al., 2022). Notably, *psaF* and *psaJ* were inserted between *psbC* and *psbB*, which has not been observed in any other FaRLiP clusters (Gan et al., 2014, 2015; Chen et al., 2019; Sheridan et al., 2020). In addition, the orientation of the phytochrome cascade genes (*rfpA*/*B*/*C*) has been reversed (Supplementary Figure 2).

Furthermore, photosystem I paralogs *psaA2, psaB2, psaL2*, and *psaI2* are missing. Although a few FaRLiP strains exist where some PSI genes are relocated to other genomic areas, e.g., *psaF2* and *psaJ2* in *Chroococcidiopsis thermalis* PCC 7203, or *psaA2*/*B2/L2/I2* in *Calothrix* sp. NIES-3974 (Gan et al., 2014, 2015; Sheridan et al., 2020), our combined bioinformatics and molecular biology approach supports this as a genuine gene loss. This represents a novel FaRLiP cluster. It would have structural implications for photoacclimation in far-red light, showing that FR photosynthesis may occur with a red-shifted PSII connected with a less red-shifted PSI or that the incorporation of chlorophyll *f* into a standard (WL) PSI might be sufficient to support far-red photosynthesis, which has been observed when expressing *chlF* in a non-FaRLiP cyanobacterium (Tros et al., 2020). Future studies using isolated PSI from FRL-grown *Chroococcidiopsis* sp. CCMEE 010 will reveal the composition of this unusual complex. Far-red paralog *psbH2* was also not detected, but it is an optional gene not present in all strains. It is possible, though highly unlikely, that some of the small (<560 bp) genes were simply missed by the genome analysis and will be revealed by future transcriptomics/proteomics studies.

It is puzzling that strains isolated from the same microenvironment, the same desert (or neither) as the FaRLiP positive CCMEE 010 (chasmoendolithic on granite, Negev desert), were FaRLiP negative in this study. Such a low FaRLiP occurrence among desert strains of *Chroococcidiopsis* suggested that this photoacclimation mechanism might not be common among rock-inhabiting cyanobacteria. Enrichment cultures may nevertheless reveal it, for instance, in samples from Negev sandstone or the Atacama Desert (Murray et al., 2022). It is possible that intense abiotic stress could result in reduced competition from other photosynthetic organisms (Lacap-Bugler et al., 2017) and, consequently, a decreased evolutionary pressure to maintain a complex acclimation response. Reduced selective pressure might also explain the unusual level of FaRLiP gene rearrangement. Synteny and gene orientation tend to be highly conserved for genes in the FaRLiP cluster, in some cases, across high-level taxonomic groups such as orders (Gan et al., 2014, 2015; Sheridan et al., 2020); changes between closely related species tend to be minor (e.g., insertions of non-FaRLiP genes or split clusters). This hints at co-regulation at a transcriptional level and suggests that a cluster showing high levels of rearrangement might, in contrast, be under relaxed selection.

The discovery of a FaRLiP-positive *Chroococcidiopsis* strain isolated from an extreme, dry environment nevertheless supports the habitability of exoplanets orbiting stars that are poor in visible light but rich in far-red/near infra-red light. In a previous study, three FaRLiP cyanobacteria, namely, *Chlorogloeopsis fritschii* PCC 6912 from thermal springs, *Chroococcidiopsis thermalis* PCC 7203 from a forest soil sample and *Synechococcus* sp. PCC 7335 from snail shells in the intertidal zone, were shown to grow under an M-dwarf simulated spectrum (Claudi et al., 2021). Although the non-FaRLiP *Synechocystis* sp. PCC 6803 also grew (by using the visible part of the simulated spectrum), it is likely that in environments more complex than unialgal cultures, oxygenic photosynthesis above 700 nm could provide a competitive advantage and therefore be more likely to evolve. Anoxygenic near-infrared photosynthesis/phototrophy would also be favored.

If far-red photosynthesis occurs on exoplanets, it might be possible to detect it. The reflectance spectrum of dried cells of *Chroococcidiopsis* sp. CCMEE 010 showed a significant shift (approximately 50 nm) of the “red edge” with respect to a non-FaRLiP *Chroococcidiopsis* strain. This might in principle serve as a diagnostic of the prevalent type of photosynthetic pigment acting on another planet, provided that it covers a large fraction of the surface (Hegde et al., 2015). In fact, dried microbial samples were reported to have higher reflectance than hydrated ones, suggesting that the reflectance signal could be more intense for exoplanets and moons drier than on Earth (Coelho et al., 2022). Nevertheless, a large percentage of planets orbiting within the habitable zone of small-mass M-class stars are predicted to be tidally locked (Barnes, 2017). With one side of the planet permanently facing the sun and intense storms caused by temperature differences, life might be restricted to the “twilight zone” and, therefore, surface signatures might be challenging to detect.

Furthermore, the habitability of planets orbiting active M-stars is placed at risk by strong UV and ionizing radiation. Thus, microbial phototrophs might take refuge under the surface, resulting in a cryptic photosynthesis, undetectable in a planetary reflectance spectrum (Cockell et al., 2009; Cockell, 2014). Nevertheless, the discovery of a FaRLiP-positive *Chroococcidiopsis* strain supports the possibility of oxygenic photosynthetic life on exoplanets. Desert strains of *Chroococcidiopsis* are resistant to UV and gamma radiation (Billi et al., 2000; Baqué et al., 2013), on a similar order of magnitude as the well-known radioresistant bacterium *Deinococcus radiodurans* (Slade and Radman, 2011). UVC-absorbing pigments such as scytonemin (Cockell, 1998), or possible alternative UV-protective mechanisms such as biofluorescence, could also be involved (O’Malley-James and Kaltenegger, 2019). Responsiveness to multiple parts of the solar spectrum is not unusual. Notably, the genes for the synthesis of UV-protectant mycosporine−like amino acids were reported to be upregulated in FRL-grown cells of *Chlorogloeopsis fritschii* PCC 6912 (Llewellyn et al., 2020). Therefore, microbial life might endure on exoplanetary surfaces highly irradiated by an M-type star long enough to complete its life cycle if it possessed radioresistance and a capacity for far-red photosynthesis.

## Data availability statement

The datasets presented in this study can be found in online repositories. The names of the repository/repositories and accession number(s) can be found in the article/Supplementary material.

## Author contributions

CM, CF, LA, VS, and RC carried out the laboratory culturing, CLSM analysis, and PCR assays. AN and LA performed the bioinformatics analysis. MS participated in the genome sequence analysis. DB and DN conceived and coordinated the study. DB, DN, and LA wrote the manuscript. AB contributed to the writing. All authors discussed the results and commented on the manuscript.

## Footnotes

1
www.nanoporetech.com

## References

[B1] Afgan E. Baker D. Batut B. van den Beek M. Bouvier D. Cech M. (2018). The galaxy platform for accessible, reproducible and collaborative biomedical analyses: 2018 update. *Nucleic Acids Res.* 46 537–544. 10.1093/nar/gky379 29790989 PMC6030816

[B2] Akutsu S. Fujinuma D. Furukawa H. Watanabe T. Ohnishi-Kameyama M. Ono H. (2011). Pigment analysis of a chlorophyll f- containing cyanobacterium strain KC1 isolated from Lake Biwa. *Photochem. Photobiol.* 33 35–40.

[B3] Antonaru L. A. Cardona T. Larkum A. W. D. Nürnberg D. J. (2020). Global distribution of a chlorophyll f cyanobacterial marker. *ISME J.* 14 2275–2287. 10.1038/s41396-020-0670-y 32457503 PMC7608106

[B4] Averina S. Velichko N. Senatskaya E. Pinevich A. (2018). Far-red light photoadaptations in aquatic cyanobacteria. *Hydrobiologia* 813 1–17. 10.1007/s10750-018-3519-x

[B5] Baqué M. Scalzi G. Rabbow E. Rettberg P. Billi D. (2013). Biofilm and planktonic lifestyles differently support the resistance of the desert cyanobacterium *Chroococcidiopsis* under space and Martian simulations. *Orig. Life Evol. Biosph.* 43 377–389. 10.1007/s11084-013-9341-6 23955666

[B6] Barnes R. (2017). Tidal locking of habitable exoplanets. *Celest. Mech. Dyn. Astr.* 129 509–536. 10.1007/s10569-017-9783-7

[B7] Behrendt L. Nielsen J. L. Sørensen S. J. Larkum A. W. Winther J. R. Kühl M. (2014). Rapid TaqMan-based quantification of chlorophyll d-containing cyanobacteria in the genus *Acaryochloris*. *Appl. Environ. Microbiol.* 80 3244–3249. 10.1128/AEM.00334-14 24632258 PMC4018932

[B8] Behrendt L. Brejnrod A. Schliep M. Sørensen S. J. Larkum A. W. Kühl M. (2015). Chlorophyll f-driven photosynthesis in a cavernous cyanobacterium. *ISME J.* 9 2108–2111. 10.1038/ismej.2015.14 25668158 PMC4542031

[B9] Billi D. Friedmann E. I. Hofer K. G. Caiola M. G. Ocampo-Friedmann R. (2000). Ionizing-radiation resistance in the desiccation-tolerant cyanobacterium *Chroococcidiopsis*. *Appl. Environ. Microbiol.* 6 1489–1492. 10.1128/AEM.66.4.1489-1492.2000 10742231 PMC92012

[B10] Chen M. Li Y. Birch D. Willows R. D. (2012). A cyanobacterium that contains chlorophyll f-a red-absorbing photopigment. *FEBS Lett.* 586 3249–3254. 10.1016/j.febslet.2012.06.045 22796191

[B11] Chen M. Hernandez-Prieto M. A. Loughlin P. C. Li Y. Willows R. D. (2019). Genome and proteome of the chlorophyll f-producing cyanobacterium Halomicronema hongdechloris: adaptative proteomic shifts under different light conditions. *BMC Genomics* 20:207. 10.1186/s12864-019-5587-3 30866821 PMC6416890

[B12] Claudi R. Alei E. Battistuzzi M. Cocola L. Erculiani M. C. Pozzer A. C. (2021). Super-earths, M dwarfs, and photosynthetic organisms: habitability in the lab. *Life* 11:10. 10.3390/life11010010 33374408 PMC7823553

[B13] Coelho L. F. Madden J. Kaltenegger L. Zinder S. Philpot W. Esquível M. G. (2022). Color catalogue of life in ice: surface biosignatures on icy worlds. *Astrobiology* 22 313–321. 10.1089/ast.2021.0008 34964651

[B14] Cockell C. S. (1998). Biological effects of high ultraviolet radiation on early earth – a theoretical evaluation. *J. Theor. Biol.* 193 717–729. 10.1006/jtbi.1998.0738 9745762

[B15] Cockell C. S. (2014). Habitable worlds with no signs of life. *Philos. Trans. A Math. Phys. Eng. Sci.* 372:20130082. 10.1098/rsta.2013.0082 24664917 PMC3982426

[B16] Cockell C. S. Kaltenegger L. Raven J. A. (2009). Cryptic photosynthesis – extrasolar planetary oxygen without a surface biological signature. *Astrobiology* 9 623–636. 10.1089/ast.2008.0273 19778274

[B17] Gan F. Zhang S. Rockwell N. C. Martin S. S. Lagarias J. C. Bryant D. A. (2014). Extensive remodeling of a cyanobacterial photosynthetic apparatus in far-red light. *Science* 345 1312–1317. 10.1126/science.1256963 25214622

[B18] Gan F. Bryant D. A. (2015). Adaptive and acclimative responses of cyanobacteria to far-red light. *Environ. Microbiol.* 17 3450–3465. 10.1111/1462-2920.12992 26234306

[B19] Gan F. Shen G. Bryant D. A. (2015). Occurrence of far-red light photo-acclimation (FaRLiP) in diverse cyanobacteria. *Life* 5 4–24. 10.3390/life5010004 25551681 PMC4390838

[B20] Goìmez-Lojero C. Leyva-Castillo L. Herrera-Salgado P. Barrera-Rojas J. Ríos-Castro E. Gutiérrez-Cirlos E. B. (2018). Leptolyngbya CCM 4, a cyanobacterium with far-red photoacclimation from Cuatro Cieìnegas Basin, Meìxico. *Photosynthetica* 56 342–353. 10.1007/s11099-018-0774-z

[B21] Gwizdala M. Lebre P. H. Maggs-Kölling G. Marais E. Cowan D. A. Krüger T. P. J. (2021). Sub-lithic photosynthesis in hot desert habitats. *Environ. Microbiol.* 23 3867–3880. 10.1111/1462-2920.15505 33817951

[B22] Harrison K. J. Crécy-Lagard V. Zallot R. (2018). Gene graphics: a genomic neighborhood data visualization web application. *Bioinformatics* 34 1406–1408. 10.1093/bioinformatics/btx793 29228171 PMC5905594

[B23] Hegde S. Kaltenegger L. (2013). Colors of extreme exo-earth environments. *Astrobiology* 13 47–56. 10.1089/ast.2012.0849 23252379

[B24] Hegde S. Paulino-Lima I. G. Kent R. Kaltenegger L. Rothschild L. (2015). Surface biosignatures of exo-earths: Remote detection of extraterrestrial life. *Proc. Natl. Acad. Sci. U.S.A*. 112, 3886–3891. 25775594 10.1073/pnas.1421237112 PMC4386386

[B25] Ho M. Y. Shen G. Canniffe D. P. Zhao C. Bryant D. A. (2016). Light-dependent chlorophyll f synthase is a highly divergent paralog of PsbA of photosystem II. *Science* 26:aaf9178. 10.1126/science.aaf9178 27386923

[B26] Johnson M. Zaretskaya I. Raytselis Y. Merezhuk Y. McGinnis S. Madden T. L. (2008). NCBI BLAST: a better web interface. *Nucleic Acids Res.* 36 W5–W9. 10.1093/nar/gkn201 18440982 PMC2447716

[B27] Kashiyama Y. Miyashita H. Ohkubo S. Ogawa N. O. Chikaraishi Y. Takano Y. (2008). Evidence of global chlorophyll d. *Science* 321:658. 10.1126/science.1158761 18669855

[B28] Kühl M. Trampe E. Mosshammer M. Johnson M. Larkum A. W. Frigaard N. U. (2020). Substantial near-infrared radiation-driven photosynthesis of chlorophyll f-containing cyanobacteria in a natural habitat. *Elife* 9:e50871. 10.7554/eLife.50871 31959282 PMC6974357

[B29] Lacap-Bugler D. C. Lee K. K. Archer S. Gillman L. N. Lau M. C. Y. Leuzinger S. (2017). Global diversity of desert hypolithic cyanobacteria. *Front. Microbiol.* 8:867. 10.3389/fmicb.2017.00867 28559886 PMC5432569

[B30] Larkum A. W. Kühl M. (2005). Chlorophyll d: the puzzle resolved. *Trends Plant Sci.* 10 355–357. 10.1016/j.tplants.2005.06.005 16019251

[B31] Llewellyn C. A. Greig C. Silkina A. Kultschar B. Hitchings M. D. Farnham G. (2020). Mycosporine-like amino acid and aromatic amino acid transcriptome response to UV and far-red light in the cyanobacterium *Chlorogloeopsis fritschii* PCC 6912. *Sci. Rep.* 10:20638. 10.1038/s41598-020-77402-6 33244119 PMC7693272

[B32] Loughlin P. Lin Y. Chen M. (2013). Chlorophyll d and *Acaryochloris marina*: current status. *Photosynth. Res.* 116 277–293. 10.1007/s11120-013-9829-y 23615924

[B33] MacGregor-Chatwin C. Nürnberg D. J. Jackson P. J. Vasilev C. Hitchcock A. Ho M. Y. (2022). Changes in supramolecular organization of cyanobacterial thylakoid membrane complexes in response to far-red light photoacclimation. *Sci. Adv.* 8:eabj4437. 10.1126/sciadv.abj4437 35138895 PMC8827656

[B34] Manning W. M. Strain H. H. (1943). Chlorophyll d: a green pigment in red algae. *J. Biol. Chem.* 151 1–19. 10.1016/S0021-9258(18)72109-1

[B35] Meslier V. Casero M. C. Dailey M. Wierzchos J. Ascaso C. Artieda O. (2018). Fundamental drivers for endolithic microbial community assemblies in the hyperarid Atacama Desert. *Environ. Microbiol.* 20 1765–1781. 10.1111/1462-2920.14106 29573365

[B36] Murray J. W. (2012). Sequence variation at the oxygen-evolving centre of photosystem II: a new class of “rogue” cyanobacterial D1 proteins. *Photosynth. Res.* 110 177–184. 10.1007/s11120-011-9714-5 22187288

[B37] Murray B. Ertekin E. Dailey M. Soulier N. T. Shen G. Bryant D. A. (2022). Adaptation of cyanobacteria to the endolithic light spectrum in hyper-arid deserts. *Microorganisms* 10:1198. 10.3390/microorganisms10061198 35744716 PMC9228357

[B38] Nürnberg D. J. Morton J. Santabarbara S. Telfer A. Joliot P. Antonaru L. A. (2018). Photochemistry beyond the red limit in chlorophyll f-containing photosystems. *Science* 360 1210–1213. 10.1126/science.aar8313 29903971

[B39] O’Malley-James J. T. Kaltenegger L. (2019). Lessons from early earth: UV surface radiation should not limit the habitability of active M star systems. *Mon. Notices Royal Astron. Soc.* 485 5598–5603. 10.1093/mnras/stz724

[B40] Ohkubo S. Miyashita H. (2017). A niche for cyanobacteria producing chlorophyll f within a microbial mat. *ISME J.* 11 2368–2378. 10.1038/ismej.2017.98 28622287 PMC5607378

[B41] Sayers E. W. Cavanaugh M. Clark K. Pruitt K. D. Schoch C. L. Sherry S. T. (2021). GenBank. *Nucleic Acids Res.* 49 D92–D96. 10.1093/nar/gkaa1023 33196830 PMC7778897

[B42] Seemann T. (2014). Prokka: rapid prokaryotic genome annotation. *Bioinformatics* 30 2068–2069. 10.1093/bioinformatics/btu153 24642063

[B43] Sheridan K. J. Duncan E. J. Eaton-Rye J. J. Summerfield T. C. (2020). The diversity and distribution of D1 proteins in cyanobacteria. *Photosynth. Res.* 145 111–128. 10.1007/s11120-020-00762-7 32556852

[B44] Sievers F. Higgins D. G. (2014). Clustal omega, accurate alignment of very large numbers of sequences. *Methods Mol. Biol.* 1079 105–116. 10.1007/978-1-62703-646-7_6 24170397

[B45] Slade D. Radman M. (2011). Oxidative stress in *Deinococcus radiodurans*. *Microbiol. Mol. Biol. Rev.* 75 133–191. 10.1128/MMBR.00015-10 21372322 PMC3063356

[B46] Smith H. D. Baqueì M. Duncan A. G. Lloyd C. R. McKay C. P. Billi D. (2014). Comparative analysis of cyanobacteria inhabiting rocks with different light transmittance in the Mojave Desert: a Mars terrestrial analogue. *Int. J. Astrobiol.* 13 271–277. 10.1017/S1473550414000056

[B47] Trampe E. Kühl M. (2016). Chlorophyll f distribution and dynamics in cyanobacterial beachrock biofilms. *J. Phycol.* 52 990–996. 10.1111/jpy.12450 27439961

[B48] Trinugroho J. P. Beèková M. Shao S. Yu J. Zhao Z. Murray J. W. (2020). Chlorophyll f synthesis by a super-rogue photosystem II complex. *Nat. Plants* 6 238–244. 10.1038/s41477-020-0616-4 32170286

[B49] Tros M. Bersanini L. Shen G. Ho M. Y. van Stokkum I. H. M. Bryant D. A. (2020). Harvesting far-red light: functional integration of chlorophyll f into photosystem I complexes of *Synechococcus* sp. PCC 7002. *Biochim. Biophys. Acta Bioenerg.* 1861:148206. 10.1016/j.bbabio.2020.148206 32305412

[B50] Uniprot C. (2021). UniProt: the universal protein knowledgebase in 2021. *Nucleic Acids Res.* 49 D480–D489. 33237286 10.1093/nar/gkaa1100 PMC7778908

[B51] Vítek P. Ascaso C. Artieda O. Casero M. A. Wierzchos J. (2017). Discovery of carotenoid red-shift in endolithic cyanobacteria from the Atacama Desert. *Sci. Rep.* 7:11116. 10.1038/s41598-017-11581-7 28894222 PMC5593868

[B52] Waterhouse A. M. Procter J. B. Martin D. M. Clamp M. Barton G. F. (2009). Jalview Version 2 – a multiple sequence alignment editor and analysis workbench. *Bioinformatics* 25 1189–1191. 10.1093/bioinformatics/btp033 19151095 PMC2672624

[B53] Wick R. R. Judd L. M. Gorrie C. L. Holt K. E. (2017). Unicycler: resolving bacterial genome assemblies from short and long sequencing reads. *PLoS Comput. Biol.* 13:e1005595. 10.1371/journal.pcbi.1005595 28594827 PMC5481147

[B54] Wierzchos J. Cámara B. de Los Ríos A. Davila A. F. Sánchez Almazo I. M. Artieda O. (2011). Microbial colonization of Ca-sulfate crusts in the hyperarid core of the Atacama Desert: implications for the search for life on Mars. *Geobiology* 9 44–60. 10.1111/j.1472-4669.2010.00254.x 20726901

[B55] Wiltbank L. B. Kehoe D. M. (2019). Diverse light responses of cyanobacteria mediated by phytochrome superfamily photoreceptors. *Nat. Rev. Microbiol.* 17 37–50. 10.1038/s41579-018-0110-4 30410070

[B56] Zhang Z.-C. Li Z.-K. Yin Y.-C. Li Y. Jia Y. Chen M. (2019). Widespread occurrence and unexpected diversity of red-shifted chlorophyll producing cyanobacteria in humid subtropical forest ecosystems. *Environ. Microbiol.* 21 1497–1510. 10.1111/1462-2920.14582 30838735

[B57] Zhao C. Gan F. Shen G. Bryant D. A. (2015). RfpA, RfpB, and RfpC are the master control elements of far-red light photoacclimation (FaRLiP). *Front. Microbiol.* 6:1303. 10.3389/fmicb.2015.01303 26635768 PMC4658448

