# Supplementary material for: Identification of far-red light acclimation in an endolithic Chroococcidiopsis strain and associated genomic features: Implications for oxygenic photosynthesis on exoplanets
Source: Front Microbiol. 2022 Aug 4;13:933404. doi: 10.3389/fmicb.2022.933404 (PMC9386421; doi:10.3389/fmicb.2022.933404)
Supplement: Supplementary file 1 [file Data_Sheet_1.doc]

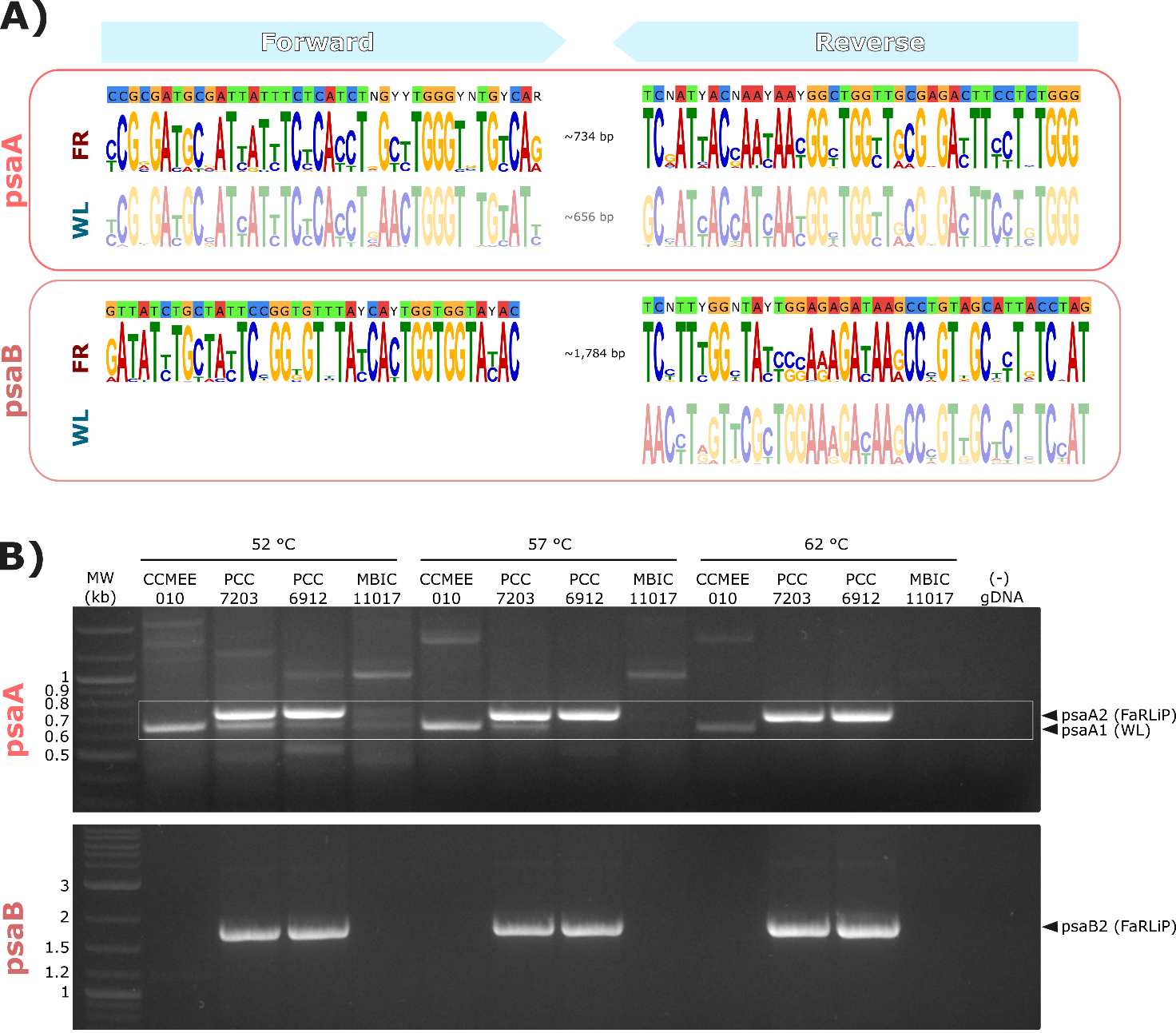
**Supplementary information**

**Figure S1** |**.** Far-red photoacclimation paralogues *psaA2* and *psaB2* are missing in strain *Chroococcidiopsis* sp. CCMEE 010.

**A)** Primer design. *psaA* and *psaB* are among the most conserved genes in photosynthesis. Far-red specific motifs (FR) that are both conserved and distinct from standard, white-light (WL) paralogues are rare and small. For *psaA*, the high sequence similarity between these FR and WL motifs highlighted the risk that the primers (coloured boxes, above the motifs) would amplify both paralogues non-specifically. However, the FaRLiP *psaA2* carries a 70-80 bp insertion, which is absent in the WL sequence, and could be used to distinguish it. In contrast, for *psaB*, although the FR region targeted by the forward primer is virtually identical to the WL one (WL motif not shown), the reverse primer carries more FR-specific bases at the selective 3’ end. Due to sequence variation, degenerate (*i.e.* variable) primers were used. Ambiguous base notation as follows: Y (C or T), R (A or G), N (all bases). Reverse primers represented as reverse complement. Figure built by using the MEME webserver (Bailey et al., 2009.

**B)** Primer testing. Primers were tested on *Chroococcidiopsis* sp. CCMEE 010, a closely-related strain (*Chroococcidiopsis thermalis* PCC 7203), and a strain in a closely-related order (the heterocyst-forming cyanobacterium *Chlorogloeopsis* sp. PCC 6912). The latter two are known to carry all FaRLiP genes. A distantly-related strain lacking FaRLiP (*Acaryochloris marina* MBIC11017) was used as a negative control. Three annealing temperatures were used, allowing high (52 °C) to low (62 °C) amounts of non-specific amplification. For *psaA* (top), at low temperatures the primers amplified both psaA2 (FaRLiP) and psaA1 (WL). However, at higher temperatures, by restricting non-specific binding, only psaA2 amplicons were recovered. For CCMEE 010, regardless of temperature, only a smaller band of ~650 bp was recovered. Sequencing confirmed it as the standard, WL-associated *psaA1*. The *psaB* primers show the absence of this FaRLiP gene in CCMEE 010 more clearly. MW – molecular weight marker (Purple 1kb Plus Ladder, NEB). Figure built with Inkscape 1.1.


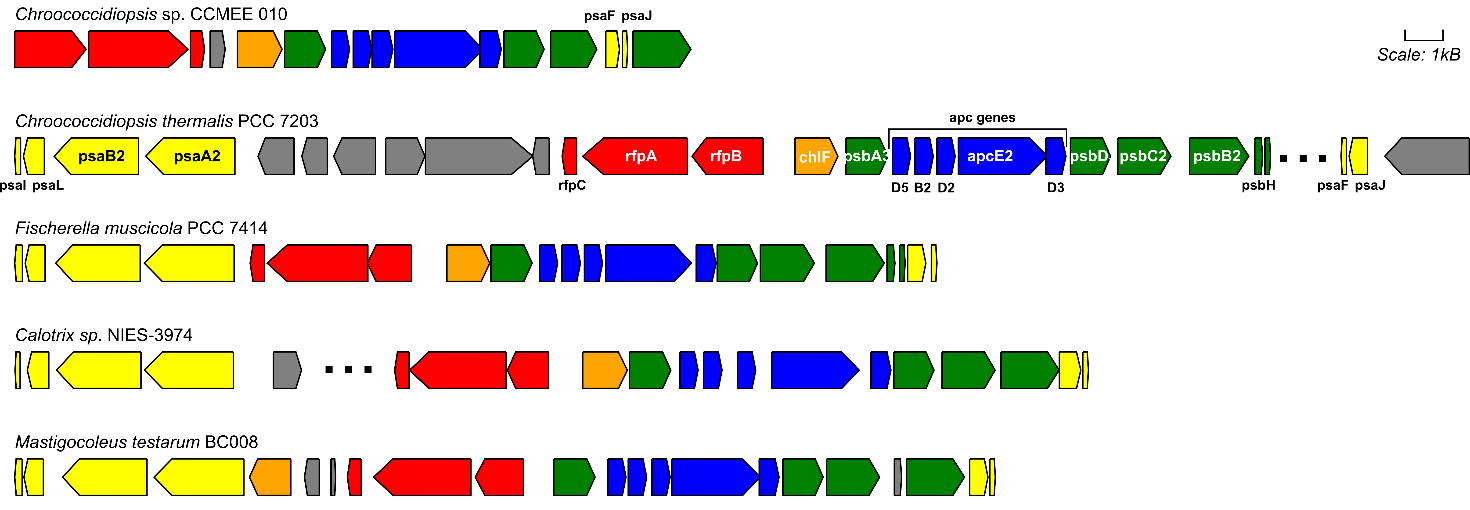


**Figure S2** |**.** *Chroococcidiopsis* sp. CCMEE 010 has a FaRLiP cluster with a distinct gene arrangement. This can be seen by comparing it with the homologous cluster in the related *Chroococcidiopsis thermalis* PCC 7203, as well as clusters from multiple heterocyst-forming cyanobacteria (the sister-clade to the Chroococcidiopsidales) (Fewer, Friedl & Büdel, 2002). The FaRLiP cluster includes paralogues of PSII genes (green), PSI (yellow), phycobilisome (blue), as well as the Chl*f* synthase (orange) and components of a regulatory signalling cascade (red). Genes with an unknown or unrelated function are shown in grey. Ellipses separate cluster fragments located in different areas of the genome. Figure build with Gene Graphics (Harrison, Crécy-Lagard & Zallot, 2018) and Inkscape 1.1, using data from Sherida*n et a*l., 2020.

| Primer^a^ | Sequence (5’ to 3’)^b^ | Degeneracy | Length | Gene |
| --- | --- | --- | --- | --- |
| f_psaA2_Chroo | CCGCGATGCGATTATTTCTCATCTNGYYTGGGYNTGYCAR | 512 | 40 | psaA2 |
| r_psaA2_Chroo | CCCAGAGGAAGTCTCGCAACCAGCCRTTRTTNGTRATNGA | 128 | 40 | psaA2 |
| f_psaA2_Chroo_t | CCGCGATGCGATTATTTCT | 0 | 19 | psaA2 |
| r_psaA2_Chroo_t | CAGAGGAAGTCTCGCAACCAG | 0 | 21 | psaA2 |
| f_psaB2_Chroo | GTTATCTGCTATTCCGGTGTTTAYCAYTGGTGGTAYAC | 8 | 38 | psaB2 |
| r_psaB2_Chroo | CTAGGTAATGCTACAGGCTTATCTCTCCARTANCCRAANGA | 64 | 41 | psaB2 |
| f_psaB2_Chroo_t | GTTATCTGCTATTCCGGTG | 0 | 19 | psaB2 |
| r_psaB2_Chroo_t | CTAGGTAATGCTACAGGCTTAT | 0 | 22 | psaB2 |
| **Table S1.** Degenerate primers used for amplifying FaRLiP-specific paralogues of *psaA* and *psaB* in *Chroococcidiopsis* and related strains. ^a^ f- forward; r – reverse. ^b^ Primers include a variable / degenerate 3’ region, and a non-variable 5’ tag. The 3’ can match a range of sequences by having positions at which bases vary (coloured highlights), thereby accounting for variation between taxonomic units. N - any base; R - A or G; Y – C or T. Each degenerate primer is therefore a collection of primers. The 5’ non-variable tag is used to improve PCR amplification, as otherwise the variable 3’ sequences might not share enough similarity to properly anneal to each other. The tags were loosely based on the genomic region just upstream of that targeted by the 3’ end, similar to the CODEHOP method (Rose, 2005). Tags were also used separately (noted with _t) as sequencing primers, and for further amplification of the PCR product. | | | | |

**Supplementary references**

Fewer, D., Friedl, T. & Büdel, B. (2002) Chroococcidiopsis and heterocyst-differentiating cyanobacteria are each other’s closest living relatives. *Molecular Phylogenetics and Evolution*. [Online] 23 (1), 82–90. Available from: doi:10.1006/mpev.2001.1075.

Harrison, K.J., Crécy-Lagard, V. De & Zallot, R. (2018) Gene Graphics: A genomic neighborhood data visualization web application. *Bioinformatics*. [Online] 34 (8), 1406–1408. Available from: doi:10.1093/bioinformatics/btx793.

Rose, T.M. (2005) CODEHOP-mediated PCR - A powerful technique for the identification and characterization of viral genomes. *Virology Journal*. [Online] 2, 1–24. Available from: doi:10.1186/1743-422X-2-20.

Sheridan, K.J., Duncan, E.J., Eaton-Rye, J.J. & Summerfield, T.C. (2020) The diversity and distribution of D1 proteins in cyanobacteria. *Photosynthesis Research*. [Online] 145 (2), 111–128. Available from: doi:10.1007/s11120-020-00762-7.
